# Changes in Periodontal Tissues With Periodontally Accelerated Orthodontics: A Systematic Review and Meta-Analysis

**DOI:** 10.7759/cureus.68795

**Published:** 2024-09-06

**Authors:** Heidy Villamil-Jaramillo, Jaime Guerrero-García, Melissa Upegui-Ramirez, Leidys H Rivera-Quiroz, Anny Vivares, Carlos M Ardila

**Affiliations:** 1 Orthodontics, Fundación Universitaria Visión de Las Américas, Medellín, COL; 2 Research Department, Faculty of Dentistry, Fundación Universitaria Visión de Las Américas, Medellín, COL; 3 Basic Sciences Department, Faculty of Dentistry, University of Antioquia, Medellín, COL

**Keywords:** corticotomy, orthodontics, periodontally accelerated orthodontics, piezocision, surgically assisted orthodontics, tomography

## Abstract

Periodontally accelerated orthodontic (PAO) therapy has been found to increase hard tissue, helping to decrease orthodontic relapse rates and improve retention capacity. The aim of this study was to synthesize available evidence on clinical and tomographic changes in periodontal tissues when using PAO techniques.

A systematic review with meta-analysis was conducted following the Preferred Reporting Items for Systematic Reviews and Meta-Analyses (PRISMA) statement and registered in the International Prospective Register of Systematic Reviews (PROSPERO). The search was carried out in PubMed, Embase, Cochrane, Web of Science, Scopus, and Google Scholar. Randomized and non-randomized clinical trials comparing PAO versus conventional orthodontics were included. Quality assessment was performed using the Downs & Black scale, and the risk of bias was assessed using the revised Cochrane risk-of-bias tool. Mean differences and 95% confidence intervals (CIs) were calculated, and the mean difference was divided by a t-test.

During the initial search, 465 studies were identified. Five articles studying 130 patients were included, which assessed both clinical and tomographic changes, along with treatment duration. PAO was administered to patients with skeletal class III in three studies, to class II patients in one study, and to individuals with dental crowding in another study. Two studies showed a moderate risk of bias, and the rest showed a low risk. The meta-analysis revealed a vestibular bone thickness increase of 0.32 mm (0.56-008; P = 0.008), a reduction of 3.12 mm (2.15-4.08; P= 0.001) in gingival retraction, and a treatment duration that was 7.07 months (8.79-5.36; P = 0.001) shorter in patients subjected to PAO compared to those undergoing conventional orthodontic treatment.

Considering the limitations of the study and acknowledging that definitive conclusions cannot be drawn, the findings suggest that treatment time decreased in patients undergoing PAO, with an increase in vestibular bone thickness and less gingival retraction observed in those undergoing this intervention.

## Introduction and background

Teeth are attached to the alveolar bone by various periodontal structures that limit their freedom of movement [1]. Dental movement induced during orthodontic treatment results from the compression of the periodontal ligament, leading to histological changes characterized by areas of bone apposition and resorption [2].

Orthodontic movements can occur in different anatomical planes, such as the vertical, transversal, and sagittal planes. Sagittal plane movement is one of the most common in corrective orthodontic treatments, often perceived by professionals as faster and simpler [1-3]. However, it requires greater care with surrounding tissues to avoid irreversible damage to the periodontium [2-4]. Orthodontic movements can occur in different anatomical planes, such as the vertical, transversal, and sagittal planes. Sagittal plane movement is one of the most common in corrective orthodontic treatments [1-3]. Despite this perceived simplicity, it is essential to exercise caution with supporting structures. Insufficient care during sagittal movements can lead to irreversible damage to the periodontium, which underscores the importance of careful planning and execution in these cases [1-4].

Understanding periodontal behavior during sagittal movement and, in general, during orthodontic treatment is crucial for achieving satisfactory results. Therefore, it is fundamental for orthodontists to be familiar with the morphology of the periodontium and the underlying bone before initiating treatment. Maintaining the integrity of periodontal tissues is often challenging, and failure to do so is associated with a significant increase in treatment times [4].

The ideal scenario is to complete treatments in less time; however, attempting to expedite conventional orthodontic treatment may not be the most appropriate approach, as the periodontium cannot withstand such forces without causing alterations at the dentoalveolar unit level [5]. Hence, one alternative gaining momentum in recent years is surgically assisted orthodontics, developed by Köle in 1959. Initially considered to violate biological principles, this technique was not widely accepted [5].

Nevertheless, in 2001, Wilcko et al. investigated the histological effects of Köle's method, introducing the concept of "regional acceleratory phenomena" (RAP). RAP refers to an invasive stimulus in hard and soft tissues, causing a local response and leading to a healing process in which tissue regeneration is faster than the physiological regeneration process [6]. Osteotomies induce increased bone remodeling of the alveolar bone due to transient and reversible alveolar demineralization, resulting in faster and less resistant dental movements. This translates to reduced treatment time and preservation of structures in the dental protection, support, and sensitivity system [5].

Osteotomy is a surgical procedure that involves elevating the full-thickness mucoperiosteal flap and making cuts or perforations in the cortical portion of the bone [4-6]. It can be performed using hand-held piercing cutting instruments such as low or high-speed rotary instruments, performing microosteoperforations, and piezoelectric instruments, referred to as piezocision; all of these are accompanied by abundant irrigation to avoid localized points of bone necrosis. Recently, elements of digital flow have been introduced to these procedures, including the incorporation of a surgical guide based on tomography to make the cuts or perforations of the corticotomy more precise [6].

Procedures of periodontally accelerated orthodontic (PAO) techniques include corticotomies, which are perforations of the cortical layer of the alveolar bone to the depth of the medullary bone [7]. PAO aims to accelerate orthodontic tooth movement through the initiation of RAP, which enhances bone remodeling and reduces the chances of gingival recession. However, it is important to distinguish PAO from periodontally accelerated osteogenic orthodontics (PAOO). While both techniques involve corticotomies, PAOO combines this procedure with bone grafting, leading to not only accelerated tooth movement but also increased alveolar bone density and volume around the corticotomy sites. The addition of bone grafts in PAOO compensates for the decreased alveolar mineralization that facilitates tooth movement, thereby providing enhanced stability and structural support at the end of the orthodontic treatment period [6-8].

Modifying the periodontal phenotype and employing surgical alternatives, as supported by several studies, can significantly enhance the long-term preservation of the periodontium in orthodontically treated patients [9-11]. These techniques optimize bone and soft tissue conditions for orthodontic movement, leading to accelerated results and shorter treatment durations [9]. Notably, PAO therapy has been shown to increase hard tissue, which contributes to reduced orthodontic relapse rates and improved retention capacity even after 10 years [10]. However, previous studies that have evaluated these techniques often focused on systematic synthesis processes and the impact on orthodontic treatment time [11], while the advantages of the technique and the clinical and radiographic changes observed were assessed using diagnostic tools that have limitations in measurement precision [12-15]. This study aimed to advance the understanding of these techniques by incorporating a comprehensive evaluation of both clinical and tomographic changes in periodontal tissues. The tomographic assessment offers a more precise and detailed evaluation of hard and soft tissue modifications, providing critical insights into the effectiveness of periodontal surgical techniques during orthodontic movements. This review, therefore, synthesized the available evidence on both clinical outcomes and tomographic changes, highlighting the novel approach of using advanced imaging techniques to better understand and enhance the preservation or augmentation of the supporting and protective tissues of the teeth.

## Review

Materials and methods

Protocol and Registration

A systematic review was conducted to synthesize scientific evidence regarding clinical and tomographic dimensional changes in periodontal tissues during sagittal movements in patients treated with periodontally accelerated orthodontics compared to traditional orthodontics. This review adhered to the Preferred Reporting Items for Systematic Reviews and Meta-Analyses (PRISMA) guidelines and was registered in the International Prospective Register of Systematic Reviews (PROSPERO) (CRD42023422819).

Eligibility Criteria

The research aimed to address the research question: "What are the clinical and tomographic dimensional changes in periodontal tissues (O) during sagittal movements in adult patients (P) treated with periodontally accelerated orthodontics in conjunction with fixed orthodontics (I) compared to traditional orthodontics (C) as reported in randomized and non-randomized clinical trials (S)?" The question followed a PICOS (participants, intervention, comparison, outcome, and study design) structure.

The inclusion criteria encompassed randomized controlled trials (RCTs) and non-randomized controlled trials (NRCTs) published until June 2024 in any language. These studies compared PAO with conventional orthodontic treatment in patients over 18 years old, evaluating clinical and computed tomography changes to quantitatively estimate changes in alveolar bone thickness. Excluded from consideration were studies involving animals, systemically compromised patients, or those with syndromes presenting craniofacial effects, as well as studies involving additional treatments to periodontal surgery or those published in repositories as projects or theses. Additionally, studies that included orthodontic therapy with aligners or invisible orthodontics, systematic reviews, letters to the editor, and narrative or topic reviews were also excluded.

Information Sources and Search Strategy

The search was conducted in MEDLINE (via PubMed), Embase, and Cochrane databases, Web of Science, Scopus, and Google Scholar, and through manual searches in relevant journals. A comprehensive electronic database search was conducted from the databases' inception until June 2024, with no language restrictions. MeSH terms and Boolean operators were used to construct the final search string: (orthodontic piezocision OR orthodontic treatment acceleration OR periodontal-facilitated orthodontics OR surgically assisted orthodontics OR periodontally accelerated orthodontics OR corticotomy) AND (gingival papilla OR periodontium OR periodontal phenotype OR gum OR alveolar bone). Searches were filtered by year and article type.

Data Collection

For the extraction and construction of a database, the following information was collected: author and year, study design, objective, age, sex, diagnoses, surgical interventions, regeneration material, and complementary diagnostic aids.

Primary outcomes included clinical and tomographic changes, considering keratinized gum width, probing depth, gingival recession, and tomographic measures such as bone density (thickness and vertical height of alveolar bone) and alveolar bone level. Treatment time was assessed as a secondary outcome.

The search, verification, review, and inclusion of potentially eligible studies were independently conducted by three reviewers. Disagreements were resolved through discussion.

Assessment of bias risk and study quality in individual studies was conducted as follows: methodological quality assessment was performed using the Downs & Black scale [16], and the risk of bias was evaluated using the revised Cochrane risk-of-bias tool for RCTs (RoB 2 tool) [17] and a tool developed to assess the risk of bias in the results of non-randomized studies that compare health effects of two or more interventions (ROBINS-I) [18]. Qualitative information synthesis involved describing findings from included articles and creating tables for study characterization and evidence summary.

Quantitative synthesis involved studies with low or moderate risk of bias to assess whether bone loss, soft tissue loss, and treatment time were greater in patients undergoing periodontally accelerated orthodontics or conventional orthodontics. A random-effects meta-analysis was conducted, considering clinical heterogeneity in anatomical references used in primary studies to measure clinical and tomographic changes.

Summary Measurements

The meta-analysis calculated mean differences and 95% confidence intervals (CIs) for clinical, tomographic changes, and treatment time, based on mean changes between time 0 and time 1 and standard deviations presented in primary studies. Global effect measures were calculated using standardized mean differences to control for heteroscedasticity in population variances that could arise from clinical heterogeneity. Standard deviation values for treatment time (values not reported in some studies) were imputed by calculating the standard error (mean difference divided by t-test) based on the p-value of the treatment time difference (reported data from the studies) in the RevMan software (Cochrane Collaboration, London, UK).

The heterogeneity of meta-analyses was tested using Cochran's Q, distributed as X2, and quantified by the I2 statistic, retaining meta-analyses with values below 70% (recommended by the Cochrane Collaboration). Sensitivity analysis was conducted by removing each study and calculating the mean difference of the remaining studies to identify any studies affecting the overall effect measure. Publication bias was visually assessed using a funnel plot. Analyses were performed using the statistical package Jamovi 2.3.28.

Results

Selection of Studies

The initial search identified 465 studies, with 102 duplicate articles and 130 addressing different topics excluded. A total of 233 articles underwent title and abstract screening, leading to the exclusion of 207. Of these, 188 addressed unrelated topics, 11 involved combined periodontal surgery with other procedures such as mini-implants or orthognathic surgery, three were clinical trials conducted on animals, two solely evaluated dental changes, one involved intervention alongside orthodontic aligners, one was a clinical trial protocol, and one was a thesis.

**Figure 1:**
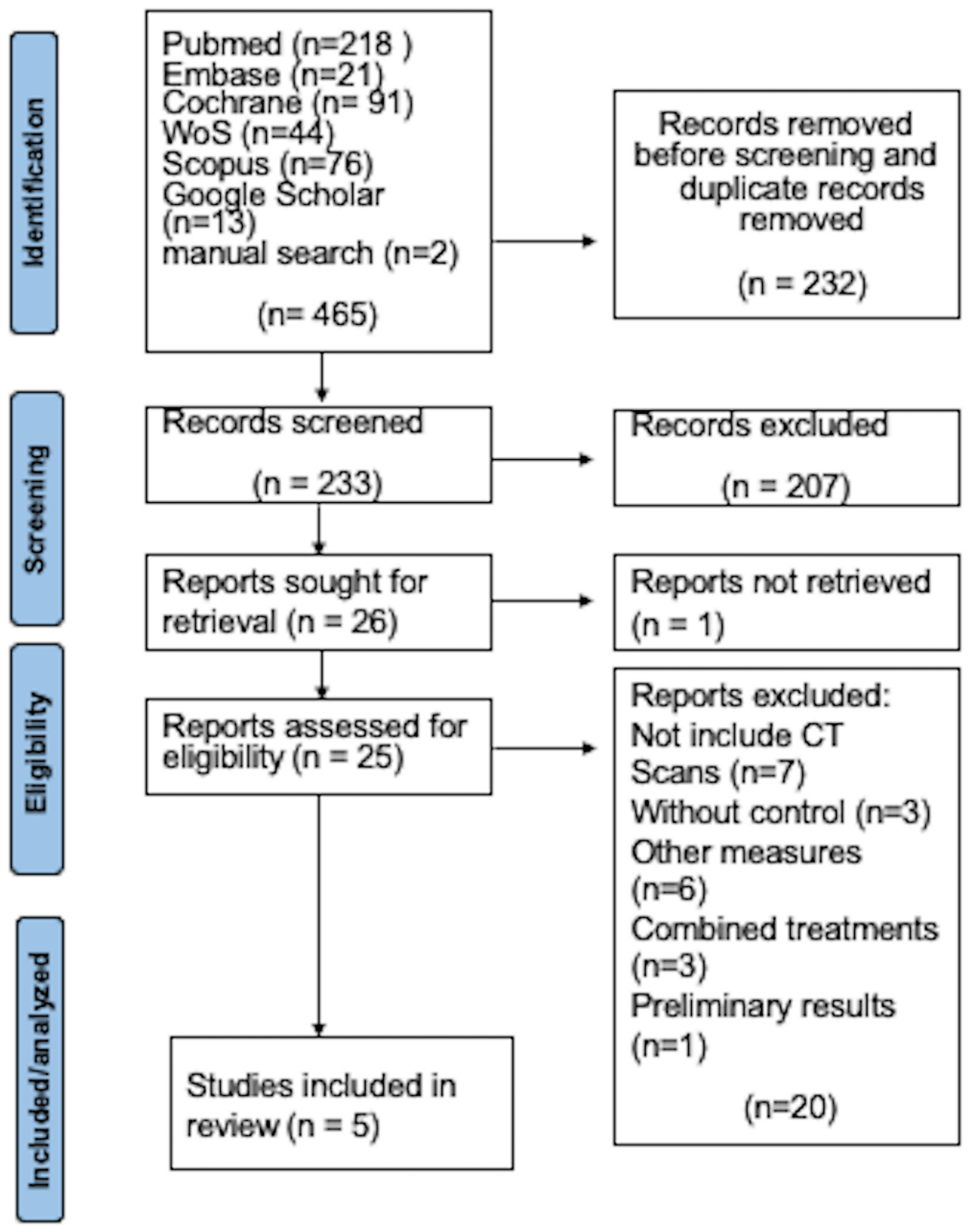
PRISMA flowchart for the article selection, screening, and inclusion process. PRISMA: Preferred Reporting Items for Systematic Reviews and Meta-Analyses.

Twenty-six articles proceeded to full-text review, but one article published in Chinese could not be retrieved. Therefore, 25 articles underwent eligibility assessment, with 18 excluded. Six did not assess changes using tomography, six evaluated other measures such as dental changes and corrections of dehiscences or bone defects, three involved combined treatments of periodontal surgery and the use of fibrin, proteins, and mini-implants, two were pre-experimental studies (lacking a control group, only assessing changes over time in a single group), one involved patients under 18 years, and one presented preliminary results of a clinical trial for which the final article could not be found. Ultimately, five articles were included in this review (Figure 1) [19-23].

Studies involving interventions on participants under 18 years and those using combined treatment with periodontal surgery were excluded because they could contribute to healing and treatment acceleration, introducing an intervention bias. Effects might be enhanced due to the combination and age, rather than the periodontal intervention itself.

Characteristics of Included Studies

Five studies were included in the review [19-23], consisting of three NRCTs and two RCTs. Four studies were conducted in Asian countries, and one study was conducted in a European country.

The total sample size across the studies was 130 patients, with a minimum of 20 patients per study and a maximum of 36 patients. The age range of participants was between 18 and 38 years. One study used a split-mouth design, while the other four studies employed patient matching. Of the two RCTs, one specified computerized randomization, and the other did not provide details on the randomization method (Table 1).

**Table 1:** Characteristics of the included studies.

Authors and year	Type of study	Country	Sample size	Comparison	Assignment
Ahn et al., 2016 [22]	Randomized clinical trial	South Korea	30	Patients	1:1
Charavet et al., 2016 [21]	Randomized clinical trial	Belgium	24	Patients	1:1
Chandra et al., 2020 [19]	Randomized clinical trial	India	20	Split mouth	1:1
Xu et al., 2020 [23]	Non-randomized clinical trial	China	20	Patients	1:1
Ma et al., 2023 [20]	Non-randomized clinical trial	China	36	Patients	1:1

In all five studies, blinding was not feasible, as patients needed to be informed about the intervention and provide informed consent. When applying the Downs and Black scale [16], scores ranged from a minimum of 19 to a maximum of 24 out of the 28 total items evaluated. Four studies were classified as having good methodological quality, and one study was classified as having excellent methodological quality (Table 2).

**Table 2:** Quality assessment (Downs and Black scale).

Authors	Randomization	Concealment	Blinding	Total	Quality
Ahn et al. [22]	Not	Not	Analysis	19/28	Good
Charavet et al. [21]	Yes unspecified	Not	Orthodontist	24/28	Excellent
Chandra et al. [19]	Yes computer	Not	Unspecified	22/28	Good
Xu et al. [23]	Not	Not	Unspecified	23/28	Good
Ma et al. [20]	Not	Not	Evaluators and analysis	21/28	Good

Risk of Bias in the Studies

**Figure 2:**
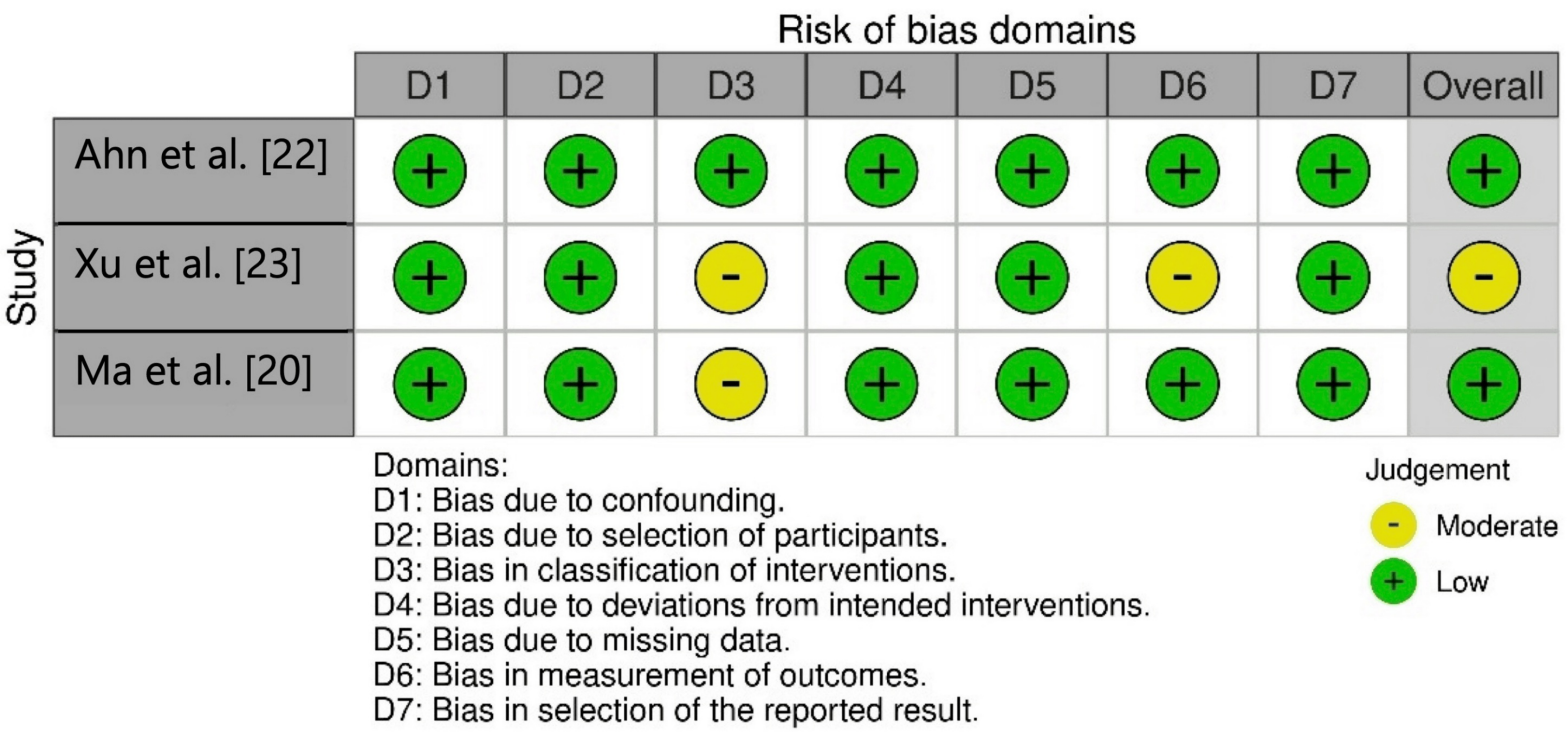
Plot of risk of bias judgments by domains of non-randomized controlled trials.

In the risk of bias analysis of the NRCTs, it was found that the study by Ahn et al. [22] exhibited a low risk of bias, while the study by Xu et al. [23] demonstrated a moderate risk of bias. This was attributed to the absence of reported matching for sex and age in the classification of interventions (D3) and the lack of evaluator blinding (D6). Regarding the study by Ma et al. [20], a moderate risk of bias was observed due to the split-mouth model design. Although matching for sex and age was not reported in the article, this information was evident in the clinical trial protocol (Figure 2).

**Figure 3:**
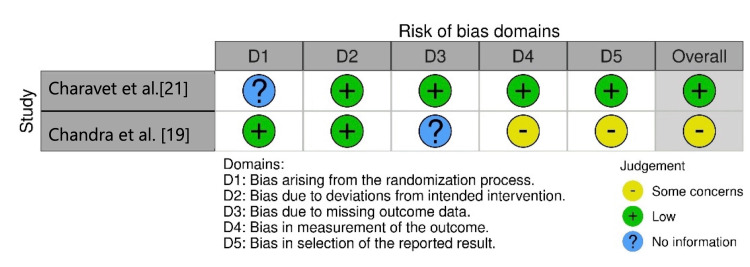
Plot of risk of bias judgments by domains of randomized controlled trials.

The study by Charavet et al. [21] exhibited a low risk of bias, whereas the study by Chandra et al. [19] showed a moderate risk of bias. The latter raised concerns due to the incomplete reporting of certain outcomes, the absence of blinding for assessors, and the lack of a protocol registration (Figure 3).

Results of Individual Studies

Patient and intervention characteristics: PAO was performed on skeletal class III patients in three studies [20-23], on class II patients in one of them [19], and on patients with dental crowding in one study [21]. The follow-up time varied, with three studies tracking patients until the completion of treatment [20-22], one monitoring every two weeks [21], and the other two following up only for six months (Table 3) [19-23].

**Table 3:** Diagnosis, surgical assistance, monitoring, and evaluations of patients in the assessed studies.

Authors	Diagnosis	With surgical assistance	Without surgical assistance	Follow-up	Evaluations
Ahn et al. [22]	Class III skeletal malocclusion	15 patients	15 patients	Until treatment is completed	2
Charavet et al. [21]	Dental crowding	12 patients	12 patients	Every 2 weeks until treatment is completed	4
Chandra et al. [19]	Class II dental malocclusion	20 patients	20 patients	6 months	4
Xu et al. [23]	Class III skeletal malocclusion	10 patients	10 patients	6 months	2

Regarding the surgical technique employed, four studies conducted corticotomies [19,20,22,23], while one utilized the piezocision technique [21]. A piezoelectric device or scalpel was used for the surgical intervention in all patients. Three studies used regeneration material post-periodontal surgery, specifically mineralized and deproteinized bovine bone or tricalcium phosphate bone substitute, all from the same commercial source [20,22,23]. These materials were used in varying amounts based on the regeneration needs, and only one study accompanied the bone graft with a regeneration membrane [20].

Concerning the intervention site, some studies targeted the upper arch while others focused on the lower arch. However, the specific location of intervention within each arch varied for each article, ranging from the left mandibular second premolar to the right mandibular second premolar [22], upper and lower central incisors [21], canine, lateral incisor, and upper second premolar on the buccal, lingual, middle, and distal surfaces [19], from the right vestibular face of the upper second premolar to the left upper second premolar [23], or from the lower right canine to the lower left canine [20].

Concerning the flap used, two studies employed full-thickness flaps [22,23], while the other studies did not report this intervention [19-21]. The vertical incisions or osteotomies performed in the five studies showed variability in depth, ranging from 2 to 3 mm. The extension of these vertical cuts also varied among studies, from 2 to 3 mm below the apex [22], 5 mm in length [21], or from 5 to 7 mm toward the apex [20], while the other two studies did not specify the millimeters of the vertical incisions (Table 4) [19,23].

**Table 4:** Surgical techniques, materials, and intervention sites in the assessed studies.

Authors	Surgical assistance				Intervention site	
	Technique	Flap	Membrane	Regeneration material	Teeth	Deep
Ahn et al. [22]	Corticotomy	Yes	Not	Mineralized and deproteinized bovine bone mineral	Second premolar to contralateral second premolar	2-3 mm
Charavet et al. [21]	Piezocision	Not	Not apply	Not performed	Upper and lower central incisors.	3 mm
Chandra et al. [19]	Corticotomy	Not	Not apply	No performed	Canine, lateral incisor, and second premolar on each side	3 mm
Xu et al. [23]	Corticotomy	Yes	Not	Tricalcium phosphate bone substitute	Second premolar to contralateral second premolar	2-3 mm
Ma et al. [20]	Corticotomy	Yes	Yes	Mineralized and deproteinized bovine bone mineral and membrane	Canine to contralateral canine	2-3 mm

Clinical Dimensional Changes

Keratinized gingiva width: None of the five articles reported changes in the width of the keratinized gingiva.

Probing depth: Three studies assessed probing depth [19,21,23] using six reference points (mesiobuccal, midbuccal, distobuccal, mesiolingual, midlingual, and distolingual on each tooth) [19], and four reference points (mesiobuccal, midbuccal, distobuccal, and mesiolingual) [21], respectively, while one study did not specify the points used [23]. In all three studies, probing depth decreased in both the experimental and control groups. However, the decrease was greater in the control group in one study [21] while in the other two, the decrease was greater in the group treated with periodontally accelerated orthodontics [19,23].

Gingival recession: Four studies reported changes in apical migration of the gingival margin [20-23]. Two studies reported gingival recession in the control group and, conversely, gingival gain in the experimental group [20,22]. One study reported gain of gingival tissue in both groups but greater in the experimental group [23], while another reported gingival recession in both the control and experimental groups, with greater recession in the group treated with periodontally accelerated orthodontics [21].

Tomographic Dimensional Changes

Width of the labial bone plate: In four out of the five studies [19,20,22,23], the experimental groups showed an increase in the width of the labial bone plate after periodontal surgical intervention, unlike the control groups. One study even reported a loss in thickness in the control group [23]. Reference points for these measurements included the apex level [20,22], or 4 mm apical to the cementoenamel junction (CEJ) [23].

Width of the lingual bone plate: Two studies reported a loss (decrease) in the width of the lingual bone plate in the experimental groups [19,20], while one study showed an increase (gain) in the width of the lingual bone plate compared to the control group [23]. Reference points used were 4 mm apical to the CEJ [19] or at the apex level [20]. The remaining studies did not specify information regarding the measurement location.

Labial and lingual alveolar height: One of the studies reported changes in alveolar height, finding a greater labial alveolar height in the experimental group compared to the control group [19].

Interproximal bone level: Only one study measured the interproximal bone [20], reporting a greater loss of the alveolar bone level in the control group than in the group where periodontally accelerated orthodontics was used.

Treatment Time

The average treatment time for the group treated with periodontally accelerated orthodontics was 6.5 months, while for the group treated with conventional orthodontics, it was 12.3 months, resulting in a halving of the treatment time for patients in the experimental group. The individually reported times in the studies were six [19], 6.39 [23], seven [21], 8.7 [22], and 15.65 [20] months for the experimental group and nine [19], 10.9 [22], 12 [23], 16 [21], and 23.3 [20] months for the control group, respectively.

Meta-analysis

**Figure 4:**
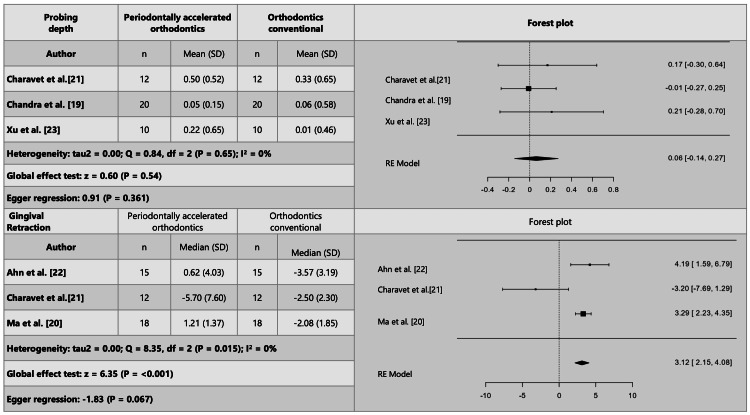
Clinical outcomes derived from the meta-analysis of periodontally accelerated orthodontics in comparison to conventional orthodontics.

For clinical changes, three studies were meta-analyzed for probing depth [19,21,23], and four for gingival recession [20-23]. However, one study was excluded due to publication bias and high heterogeneity [23], and although another study fell outside the funnel plot [21], Egger's regression indicated no publication bias with the remaining three studies that were meta-analyzed [20-22]. It was concluded that probing depth does not show significant differences when using either of the two techniques, whether periodontally accelerated orthodontics or conventional orthodontics. However, gingival recession is, on average, 3.12 mm (2.15-4.08) (P = 0.001) less when periodontally accelerated orthodontics is performed (Figure 4).

**Figure 5:**
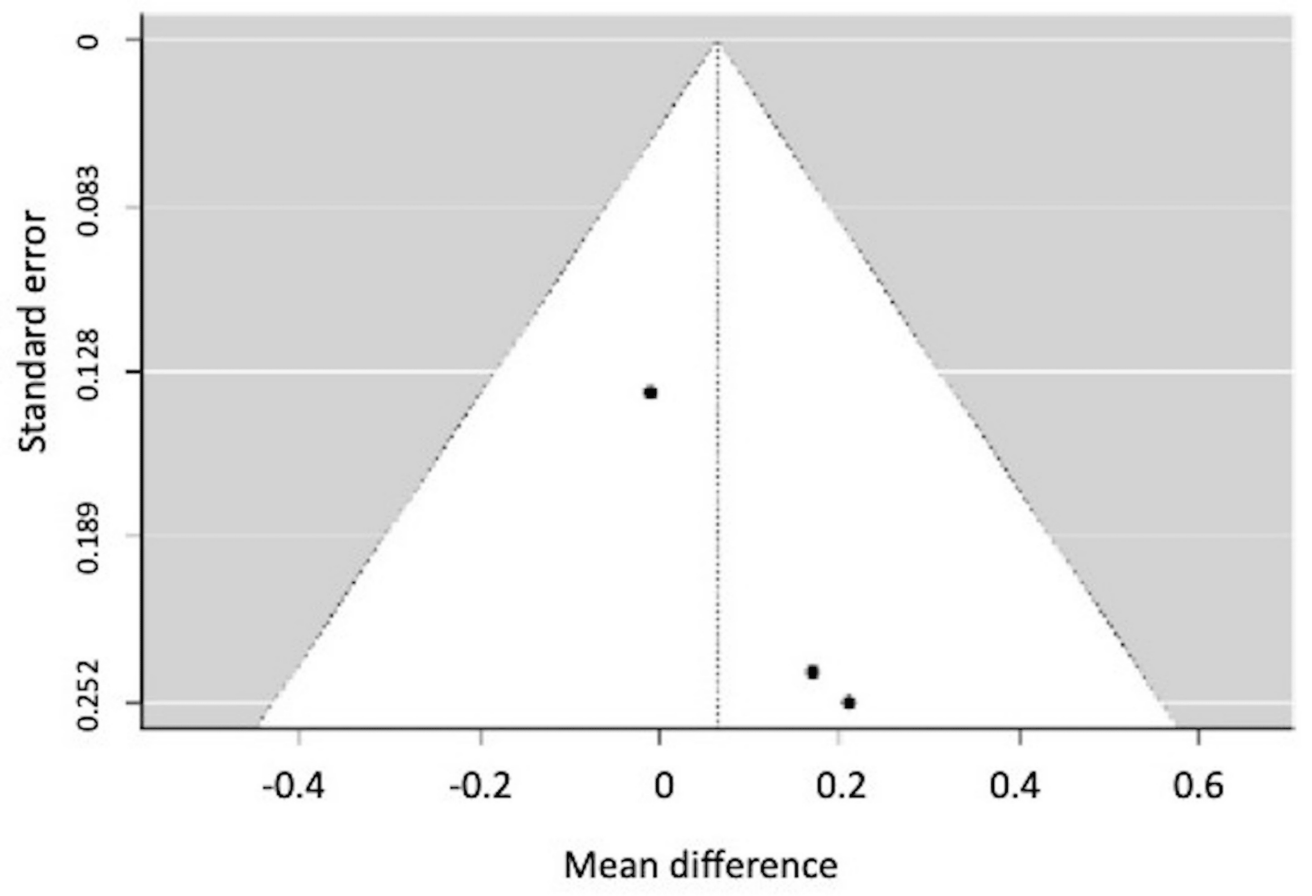
Funnel plot for probing depth.

**Figure 6:**
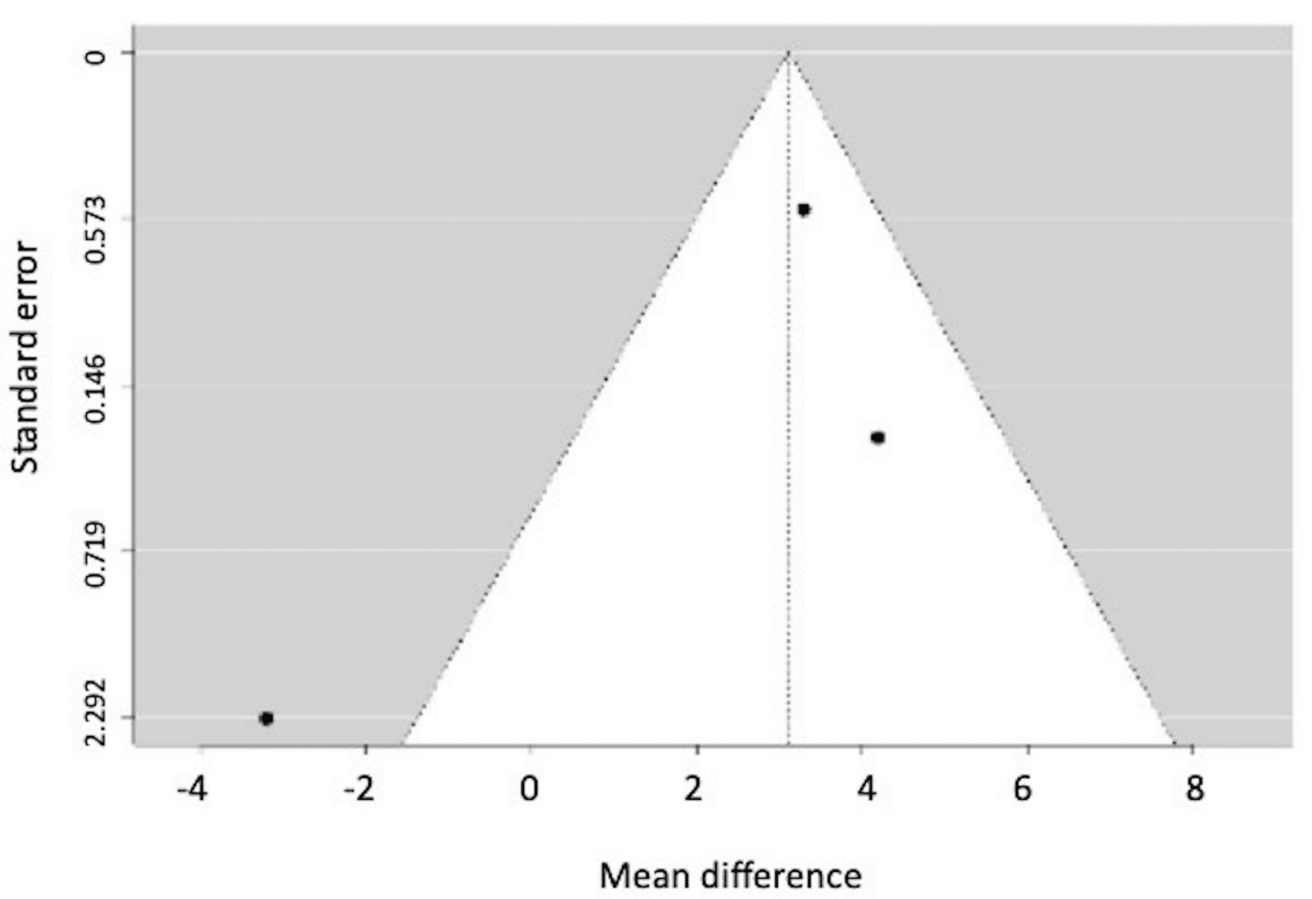
Funnel plot for gingival retraction.

Figures 5, 6 show the funnel plots for probing depth and gingival retraction.

**Figure 7:**
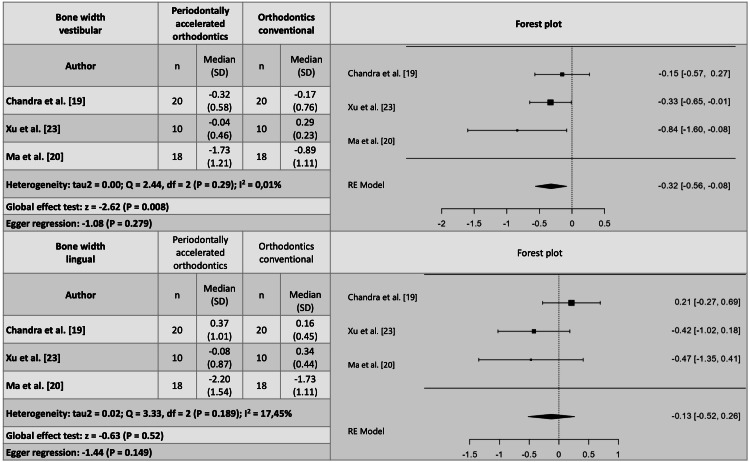
Tomographic changes derived from the meta-analysis of periodontally accelerated orthodontics compared to conventional orthodontics.

Regarding tomographic changes, four studies were meta-analyzed for the width of the vestibular bone plate [19,20,22,23]. However, one study had to be eliminated because the p-value of Egger's regression indicated publication bias [22]. Only three studies remained in the meta-analysis, the same studies that reported evaluation of the lingual bone plate and were meta-analyzed [19,20,23]. It was evidenced that patients treated with periodontally accelerated orthodontics gain an average of 0.32 mm (0.56-008) (P = 0.008) in thickness of the vestibular bone plate, but the lingual bone gain was not different between those treated with periodontally accelerated orthodontics and those treated with conventional orthodontics. It is important to clarify that the meta-analysis evaluated a gain in bone thickness, not loss. In Figure 3, the diamond is positioned on the side of the PAOO, indicating a negative global effect measure because it is on the side of the intervention. This suggests that bone thickness was gained with PAOO (Figure 7).

**Figure 8:**
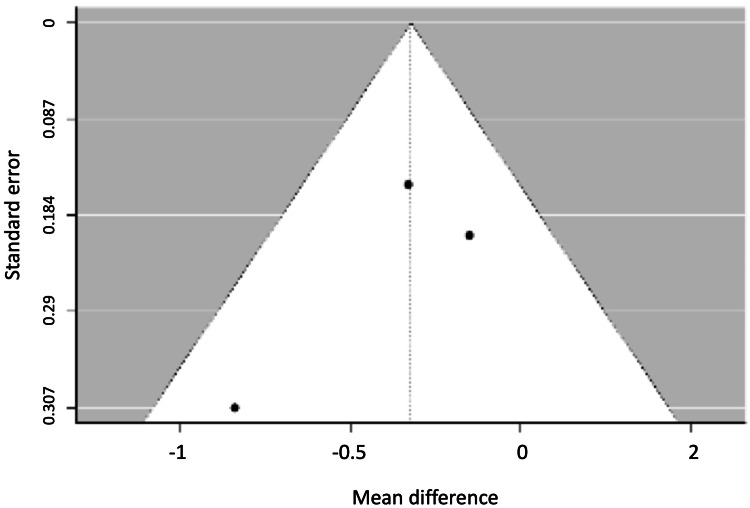
Funnel plot for the width of the vestibular bone plate.

**Figure 9:**
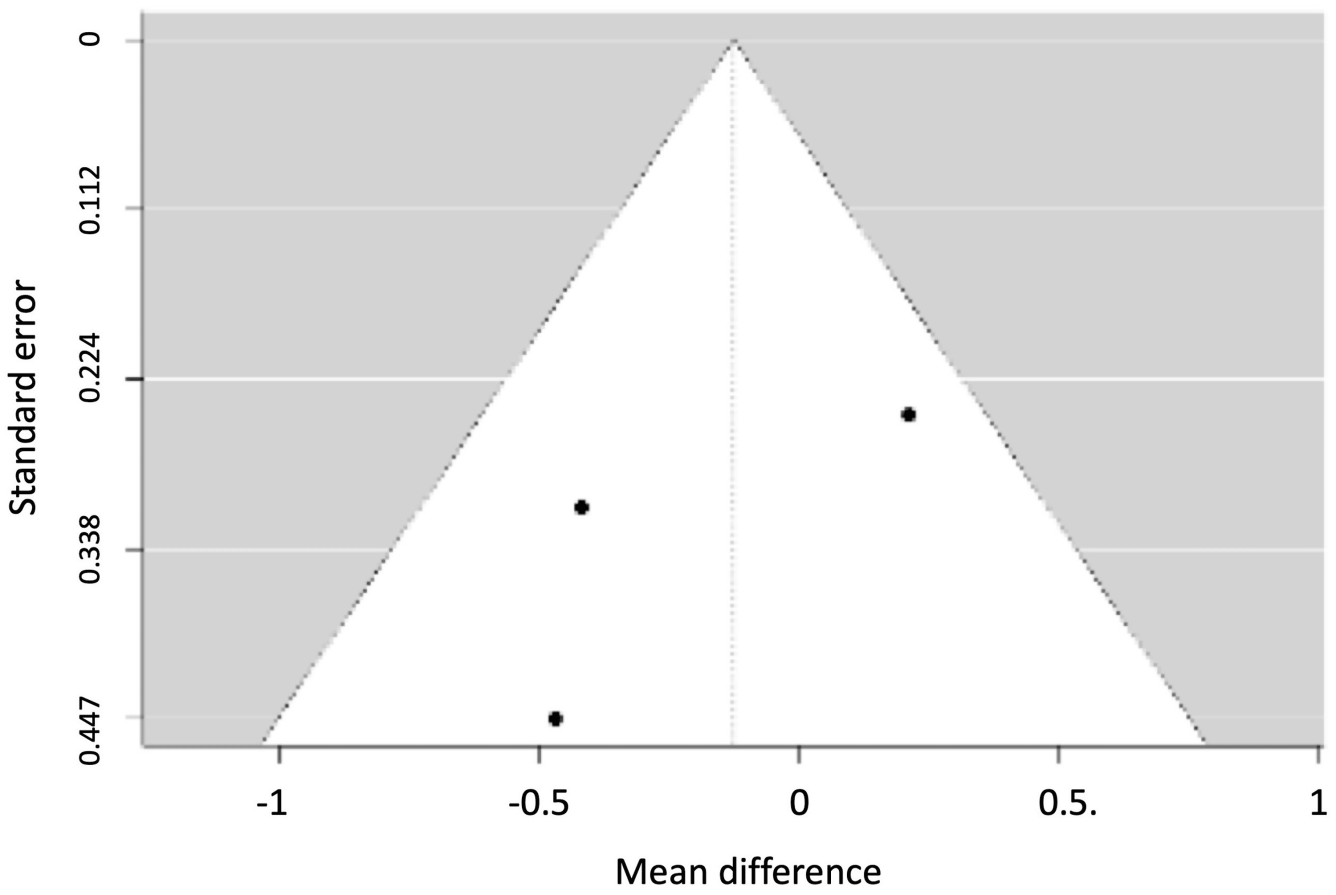
Funnel plot for the width of the lingual bone plate.

Figures 8, 9 show the funnel plots for the width of the vestibular and lingual bone plates.

**Figure 10:**
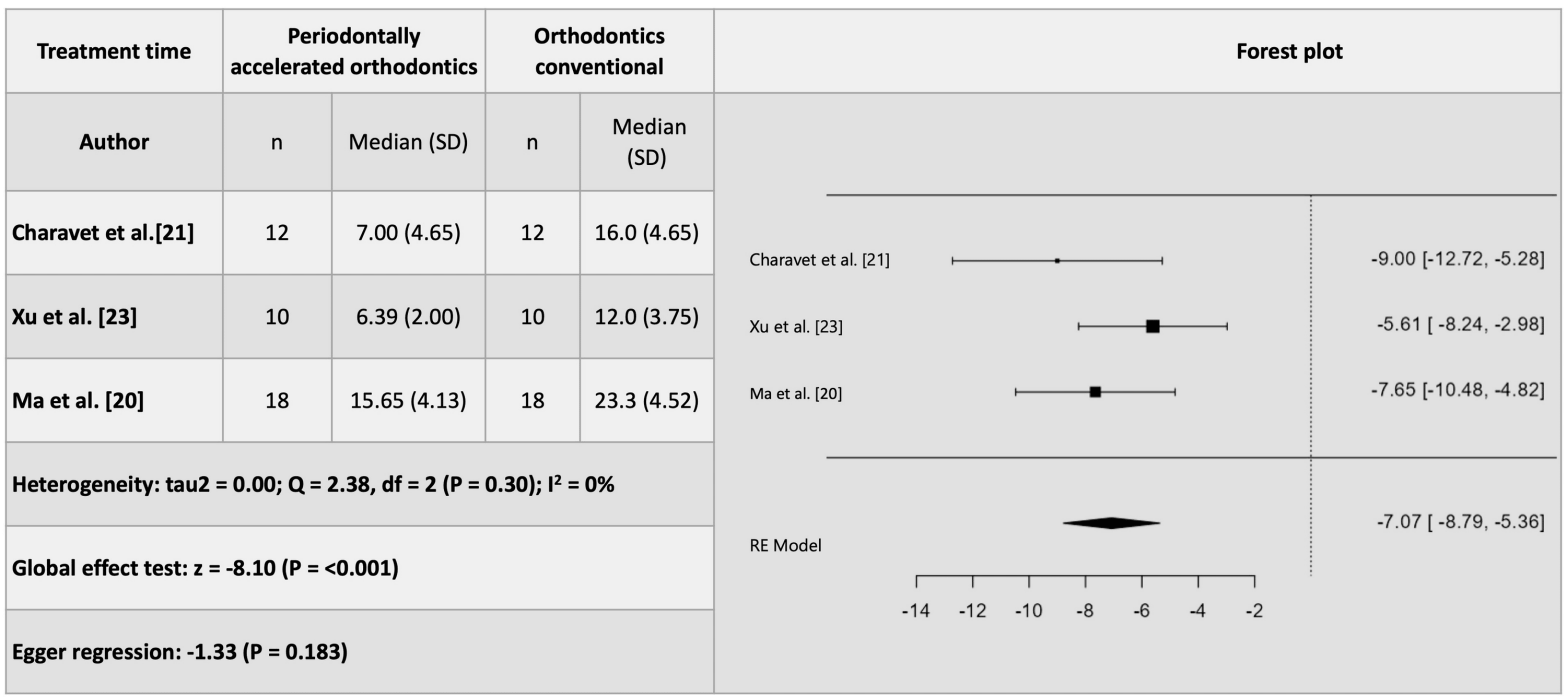
Treatment time variations identified in the meta-analysis of periodontally accelerated orthodontics versus conventional orthodontics.

Regarding the treatment time (secondary outcome), all five studies included in the review were included in the meta-analysis, but two studies [19,22] were excluded due to publication bias and an increase in meta-analysis heterogeneity [19,22]. The included studies indicate that the difference in treatment time is 7.07 months (8.79-5.36; P = 0.001) (Figure 10), with treatment being faster in those undergoing periodontally accelerated orthodontics than in patients treated with conventional orthodontics [20,21,23].

**Figure 11:**
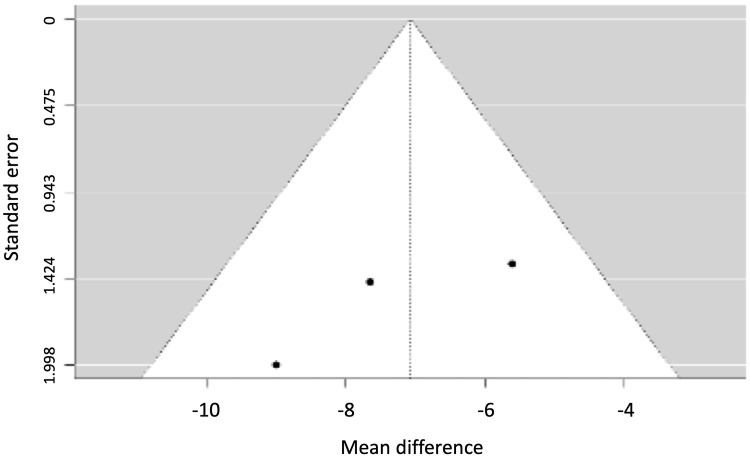
Funnel plot for treatment time.

Figure 11 shows the funnel plot for treatment time.

Discussion

This study evaluated clinical changes such as probing depth, gingival recession, and the width of keratinized gingiva. Probing depth did not differ significantly in patients treated with PAO compared to those treated with conventional orthodontics. Gingival recession was lower in the group treated with PAO, while the width of keratinized gingiva was not assessed in any of the studies. Additionally, tomographic changes were assessed in terms of vestibular and lingual bone width, revealing that vestibular bone width is greater in patients treated with periodontally accelerated orthodontics, with no significant differences in lingual bone width. As a secondary outcome, treatment time was significantly shorter in patients undergoing PAO.

During the study's development, well-conducted clinical trials were identified. Still, they evaluated bone changes without tomography or assessed treatments that, in addition to corticotomy, included the use of platelet-rich plasma, fibrin, laser, medications, or other components that, combined with the technique, could accelerate results. These were excluded because the outcome could be influenced using these elements rather than periodontal surgery, as the use of pharmacological agents such as vitamin D, growth hormone, and physical stimuli like vibrations, electromagnetic fields, and even low-level laser therapy has been shown to accelerate orthodontic movement through changes in bone metabolism [24-26].

Most studies included in this review originated from research conducted in the Asian continent, which aligns with other research indicating that most clinical trials addressing the use of corticotomy in orthodontics are carried out in Asia [27]. Asian countries are known for being transparent in reporting the results of their research [28]. Over the last two decades, there has been a notable increase in the scientific contribution of Asian and South American countries to research on corticotomy-assisted orthodontics [27].

The included studies used different terms to designate techniques that are part of the corticotomy classification. Clarifying conceptual differences is relevant. Corticotomy is a surgical procedure involving cuts, perforations, or direct mechanical alterations to the cortical bone without affecting the medullary bone. Piezocision involves cortical incisions through soft tissue with an irrigation-abundant tip. Microosteoperforations (MOP) are small perforations around teeth to be moved by orthodontics [29]. The term accelerated periodontally osteogenic orthodontics is also used, wherein the same corticotomy technique is applied, but in PAOO, a bone graft is always used to increase bone thickness, providing greater stability after orthodontic treatment [30].

Regarding clinical dimensional changes, the included studies did not report measures and changes in the width of keratinized gingiva, which was considered a relevant measure for the study. As mentioned by Coatoam et al. [31], orthodontics is a treatment that favors the width of keratinized gingiva; therefore, no orthodontic intervention should affect this clinical parameter.

Regarding probing depth and gingival recession measurements, heterogeneity was observed due to variations in the inclusion of these clinical parameters across studies. Furthermore, among studies that did report these parameters, different reference standards were utilized, leading to diverse results in some evaluations.

Probing depth was greater in one study for the group treated with conventional orthodontics and in two studies for the group treated with PAO, although this difference was not significant. This result may be associated with orthodontic therapy itself and not with periodontal surgical intervention. It has been mentioned that orthodontic treatments can affect the balance of oral microbiota, increasing plaque retention, causing gingival inflammation, bleeding on probing, and consequent periodontal disease [32].

Regarding gingival recession, qualitative synthesis reported positive and negative changes in both non-intervened and surgically intervened patients, but quantitative synthesis showed greater recession in patients treated with conventional orthodontics. In line with this, some studies following patients treated only with conventional orthodontics found inconsistencies, as some claim that it causes gingival recessions, while others indicate the opposite [33]. Amit et al. found that vestibular movements contribute to the development of gingival recessions [34], as movements that do not go beyond the bone table have a very low risk of developing gingival recessions, and it is only when the tooth is moved out of its cortical bone that there is a risk of bone dehiscence and, therefore, the development of gingival recessions [35]. However, studies evaluating the impact of PAO on periodontal soft tissues find that it does not change the level of the gingival margin [36].

Regarding tomographic findings, the studies by Wang et al. [37] and Coscia et al. [38] align with the findings of this review. These authors identified favorable dimensional changes in vestibular bone width in patients subjected to surgically facilitated orthodontic procedures. Concerning lingual bone width, the findings also align with the results found by other authors, who have shown that corticotomies accelerate the orthodontic process but generally do not have a significant impact on lingual bone tables [35-38]. It is indicated that this is due to the location of the cuts, as they are generally made in the frontal part of the alveolar bone in the portion of the bone surrounding the roots of the teeth, so the changes do not directly affect the lingual bone table, as it is not directly involved in the bone remodeling process following surgery.

However, even though the surgical technique is primarily performed on the vestibular bone when accompanied by orthodontic treatment, as indicated by Lund et al. [39], changes occur in all anatomical dimensions of dental and support tissues; therefore, it is necessary not only to evaluate the thickness of the bone table but also to assess the height and level of lingual and interproximal bone.

As for treatment time, this review corroborates findings in previous studies describing that periodontally accelerated orthodontics significantly reduces treatment time [32,40].

Several candidate articles for inclusion in the review were excluded for using combined techniques, meaning they employed periodontally accelerated orthodontics along with other techniques that could expedite healing and orthodontic movement. While these articles contained valuable information to enrich the present investigation, they could also bias the results. Additionally, some excluded studies focused solely on the tomographic evaluation of orthodontic treatment acceleration through corticotomy, neglecting clinical changes in the periodontium, thus not comprehensively addressing the effects on hard and soft tissues. Furthermore, several articles only quantified the duration of orthodontic treatments with or without this technique, leaving an incomplete understanding of the results and risks associated with the technique at the periodontal level.

On the other hand, the studies included in the review focused on a specific age group, such as adolescent or young adult patients, limiting the generalization of results to broader age groups. Additionally, some articles did not report methods of matching, blinding, or concealment in their methodologies, and their results did not include all relevant measures of hard and soft tissues, preventing them from being included in the meta-analysis. Additionally, the lack of long-term follow-up and small sample sizes were identified in the studies; therefore, the results of this review should be interpreted with caution. Due to the aforementioned factors, it is not possible to generalize these findings or extrapolate them to provide recommendations for approaching patients in the clinical setting.

It is recommended to conduct a systematic review that includes combined treatments, where not only corticotomy plus orthodontics techniques are evaluated, but also other treatments that can provide a comprehensive view of this topic. This is because evidence in some cases is insufficient to answer unresolved research questions.

Moreover, gaps in the evidence found were identified. Primary source studies need to specify the study type, and precise processes carried out should be reported. This information helps reduce the risks of biases. Therefore, efforts should be made to encourage the conduct of more randomized controlled clinical trials, as this reduces allocation bias. Additionally, it is recommended to always include the evaluation of changes in soft tissues.

Efforts should also be made to standardize the methods of performing periodontally accelerated orthodontics, as well as the terms used to refer to the technique.

## Conclusions

Considering the limitations of the study, the findings suggest that the probing depth and the width of the lingual alveolar bone are not different in patients treated with periodontally accelerated orthodontics compared to those treated with conventional orthodontics; however, periodontally accelerated orthodontics does achieve less gingival retraction, gain in the vestibular alveolar bone, and a shorter treatment time.
